# Modulation of intestinal TLR4 expression in infants with neonatal opioid withdrawal syndrome

**DOI:** 10.1038/s41372-023-01859-2

**Published:** 2023-12-27

**Authors:** Rebecca C. Barnett, Angela N. Lewis, Qingqing Gong, Deborah L. Preston, Lauren C. Frazer, Joseph W. Werthammer, Misty Good

**Affiliations:** 1 Department of Pediatrics, Marshall University, Joan C. Edwards School of Medicine, Huntington, WV, USA; 2 Division of Neonatal-Perinatal Medicine, Department of Pediatrics, Saint Louis University, Cardinal Glennon Children’s Hospital, St. Louis, MO, USA; 3 Division of Newborn Medicine, Department of Pediatrics, Washington University School of Medicine, St. Louis Children’s Hospital, St. Louis, MO, USA; 4 Present address: Division of Neonatal-Perinatal Medicine, Department of Pediatrics, University of North Carolina Children’s Hospital, Chapel Hill, NC, USA

## Abstract

**OBJECTIVE:**

Neonatal Opioid Withdrawal Syndrome (NOWS) has been associated with the development of necrotizing enterocolitis (NEC) in term and late-preterm neonates. In this study, we used stool gene expression to determine if an increase in baseline inflammation in the intestine of infants with NOWS is associated with these findings.

**STUDY DESIGN:**

Stool samples were prospectively collected between days 1–3 and days 4–9 after delivery for opioid-exposed (*n* = 9) or non-exposed neonates (*n* = 8). Stool gene expression for *TLR4* and *HMGB1* was determined via real-time PCR.

**RESULTS:**

*TLR4* expression was higher in the stool of the non-exposed group in both time periods, between days 1–3 (*P* < 0.0001) and days 4–9 (*P* < 0.05) after delivery. No significant difference in *HMGB1* expression was found at either time point *(P* > 0.05).

**CONCLUSION:**

These findings point to an important interplay between opioid exposure and/or NOWS and the inflammatory milieu of the neonatal intestine.

## INTRODUCTION

Opioid use and related deaths have dramatically increased in the United States since the 1990s [1]. Correspondingly, there has been a significant increase in cases of Neonatal Abstinence Syndrome, more recently termed Neonatal Opioid Withdrawal Syndrome (NOWS), associated with maternal opioid use during pregnancy [2, 3]. NOWS is characterized by a variety of neurologic and gastrointestinal symptoms. These include irritability, hypertonicity, poor feeding, loose stools, and difficulty sleeping, which can require opioid administration to reduce symptomatology [4]. Significant long-term neurobehavioral consequences of in utero opioid exposure have also been documented [2]. Additionally, we previously reported, in a single-center retrospective study, that in a small cohort of infants older than 35 weeks gestation, NOWS is associated with an increased risk of the intestinal disease necrotizing enterocolitis (NEC) [5].

NEC is a gastrointestinal disorder that primarily occurs in preterm neonates and has a complex pathophysiology culminating in an inflammatory response that can lead to irreversible intestinal injury [6, 7]. Risk factors for NEC include preterm delivery, low birth weight, antibiotic exposure, and lack of breast milk feedings [6, 8, 9]. Despite its association with extreme prematurity, an increased risk of NEC has been reported in late-preterm and term neonates with congenital heart disease [10] as well as in small observational studies in the setting of NOWS [5, 11]. This association of NEC with in utero opioid exposure or NOWS was not detected in a large retrospective study of >100,000 pregnant women in Tennessee that focused on outcomes for opioid-exposed neonates [12]. Thus, additional studies are needed to clarify if there are specific healthcare settings and populations of infants where NOWS is associated with an increased risk of NEC. Furthermore, mechanistic data is needed to determine if there is a causative role for in utero opioid exposure and/or NOWS in the pathogenesis of NEC.

Mechanisms implicated in the pathophysiology of NEC, which have been primarily studied in the setting of prematurity, include intestinal microbial dysbiosis, hypoxia, intestinal epithelial immaturity, and immune hyperactivation [6, 7, 13, 14]. For example, high mobility group box 1 (HMGB1) is a polypeptide released from immune cells and induces an inflammatory response by directly acting on pattern recognition receptors, including the innate immune receptor Toll-like receptor 4 (TLR4) [15, 16]. TLR4 is the receptor for lipopolysaccharide (LPS) expressed by Gram-negative bacteria, and its activation on immune and non-immune cells leads to a powerful inflammatory cascade [17–19]. Both HMGB1 and TLR4 are upregulated during NEC and have been implicated in intestinal inflammation and disease pathogenesis [14, 20–24].

In seeking to determine the potential mechanisms involved in our previously reported association between NEC and NOWS in late-preterm and full-term neonates [5], we prospectively measured the expression of *HMGB1* and *TLR4* in the stool of neonates with NOWS and controls. We hypothesized that *HMGB1* and *TLR4* expression would be increased in the stool of neonates with NOWS, as a reflection of a heightened intestinal inflammatory milieu.

## MATERIALS/SUBJECTS AND METHODS

### Population and sampling

A prospective cohort study of infants born at Hoops Family Children’s Hospital (Huntington, WV, USA) was performed from August 2020 until June 2021 after approval by the Marshall University Institutional Review Board. Inclusion criteria were a gestational age of at least 36 weeks and exclusive formula feeding. Exclusion criteria included an Apgar score of less than five at 5 min, receipt of any breast milk feeds, congenital anomalies present at birth, and concern for sepsis, respiratory distress, or other health complications. Infants who met the study criteria were recruited and enrolled after signed parental consent was obtained. Informed consent was obtained for all enrolled patients.

A urine toxicology screen (Utox) was performed for all patients upon admission for delivery at Cabell Huntington Hospital (Huntington, WV, USA). At delivery, umbilical cord tissue toxicology screening was performed for any patient with a positive toxicology screen on admission for delivery, a history of a positive toxicology screen during pregnancy, or a history of opioid or illegal drug use. All opioid-exposed infants were monitored for a minimum of five days of observation for the development of NOWS.

Stool samples utilized in this study were collected for neonates in the opioid-exposed group between days 1 and 3 and days 4 and 9 after delivery. For the non-opioid-exposed infants, a stool sample was collected between days 1 and 3 after delivery. After discharge from the hospital, the parents of the non-opioid-exposed infants were given a bag with stool collection instructions and an identifying sticker to place on a diaper for the second collection, which occurred between days 4 and 8 after delivery. Samples were subsequently brought to the infant’s Marshall Pediatrics newborn follow-up visit.

Stool samples were collected from non-absorbent diapers, homogenized by stirring, aliquoted into microcentrifuge tubes, and stored at −80°C. Infants without adequate stool samples within both time frames described above were excluded from further analysis. Deidentified stool samples were subsequently sent to Washington University School of Medicine in St. Louis, MO, USA for analysis.

### RNA isolation and PCR

RNA was isolated from ~200–300 mg of each stool sample using the Quick-RNA Fecal/Soil Microbe Microprep Kit (Zymo Research, Irvine, CA, USA) per the manufacturer’s instructions. Real-time PCR was performed as in Mihi et al. [14] *TLR4* and *HMGB1* gene expression were normalized to the housekeeping gene *RPL0*, and relative expression was reported as the 2^−ΔΔCT^.

### Data collection and statistical analysis

Baseline demographic data, including maternal age, sex, gestational age, birth weight, Apgar scores, maternal urine toxicology results, and umbilical cord toxicology results, were collected, de-identified, and stored in a REDCap database. Significant differences in baseline characteristics were determined using a Two-tailed Mann-Whitney *U* test or a Two-sided Fisher’s Exact test. A Two-tailed Mann-Whitney *U* test was used to compare relative gene expression between groups. All statistical analyses were done using GraphPad Prism, version 9.3.1, by GraphPad Software (La Jolla, CA, USA).

## RESULTS

**Fig. 1:**
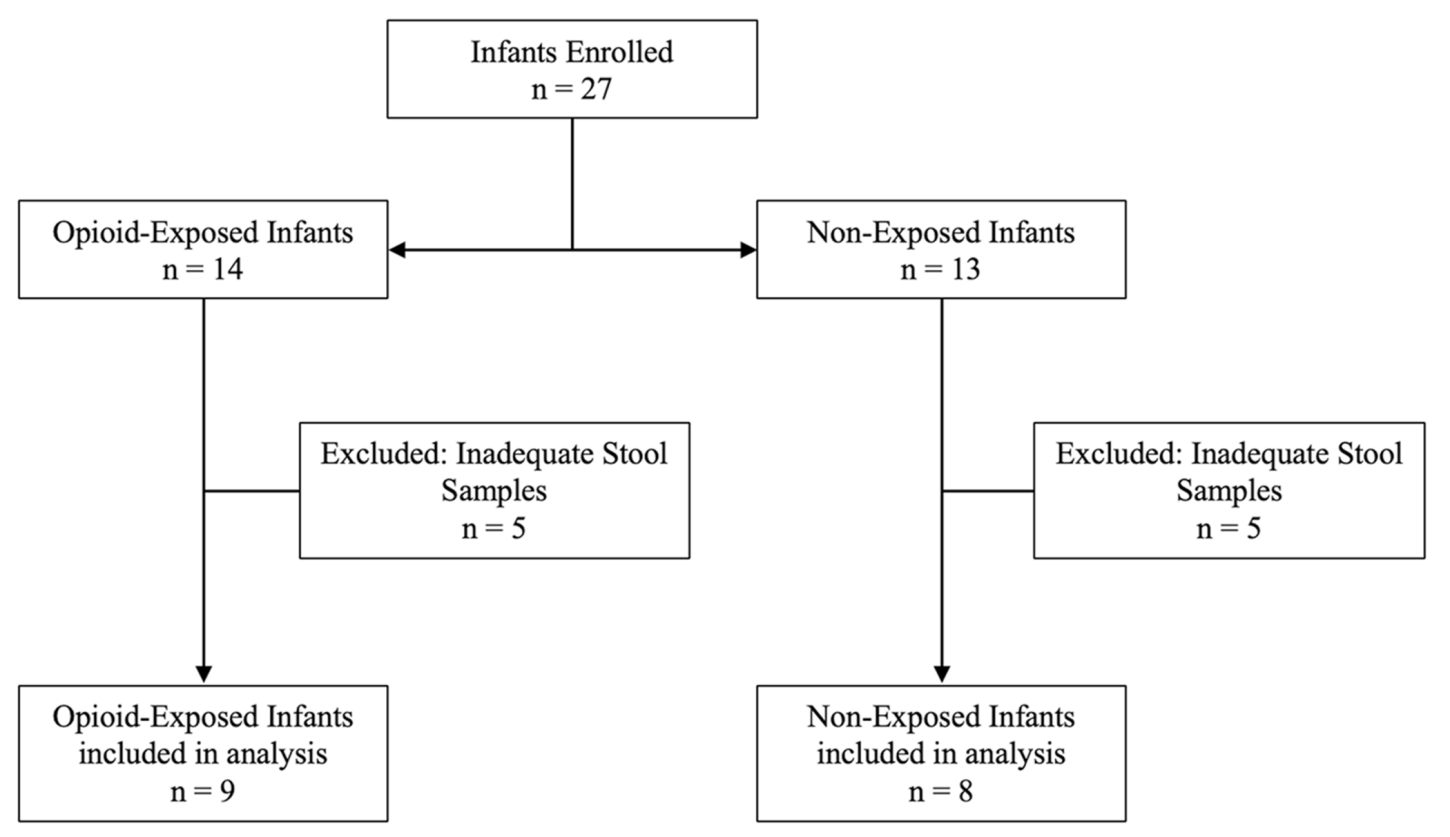
Participant enrollment and inclusion. A total of 27 infants were enrolled in the study, including 14 opioid-exposed and 13 non-exposed controls. Only infants with adequate quantities of stool on days 1–3 and days 4–9 were included in the final study, which resulted in five infants in each group being excluded from further analysis.

Twenty-seven infants were prospectively enrolled in this study (Fig. 1). Ten infants were excluded due to inadequate stool specimens (i.e., unable to be collected or of insufficient quantity). In total, nine opioid-exposed infants and eight non-exposed control infants were included in the final analysis. A comparison of the baseline characteristics of the study population (Table 1) was performed and revealed no significant differences between the groups for maternal age at delivery, sex, or birth weight. Gestational age at delivery was statistically (*P* = 0.04) but not physiologically significant, with a difference of 1 week in the mean gestational age for the groups (Table 1). The results of the maternal Utox and umbilical cord tissue toxicology screens are summarized in Table 2. Based on these results, we found that opioid exposure for the cohort included seven infants exposed to buprenorphine, one to methadone alone, and one to methadone and heroin. Toxicology screening also revealed polysubstance exposure, including tetrahydrocannabinol (THC) and cocaine, as outlined in Table 2. The maternal Utox for one infant in the control group was positive for THC. Eight of the nine opioid-exposed infants were pharmacologically treated for NOWS, as indicated in Table 2.

**Fig. 2:**
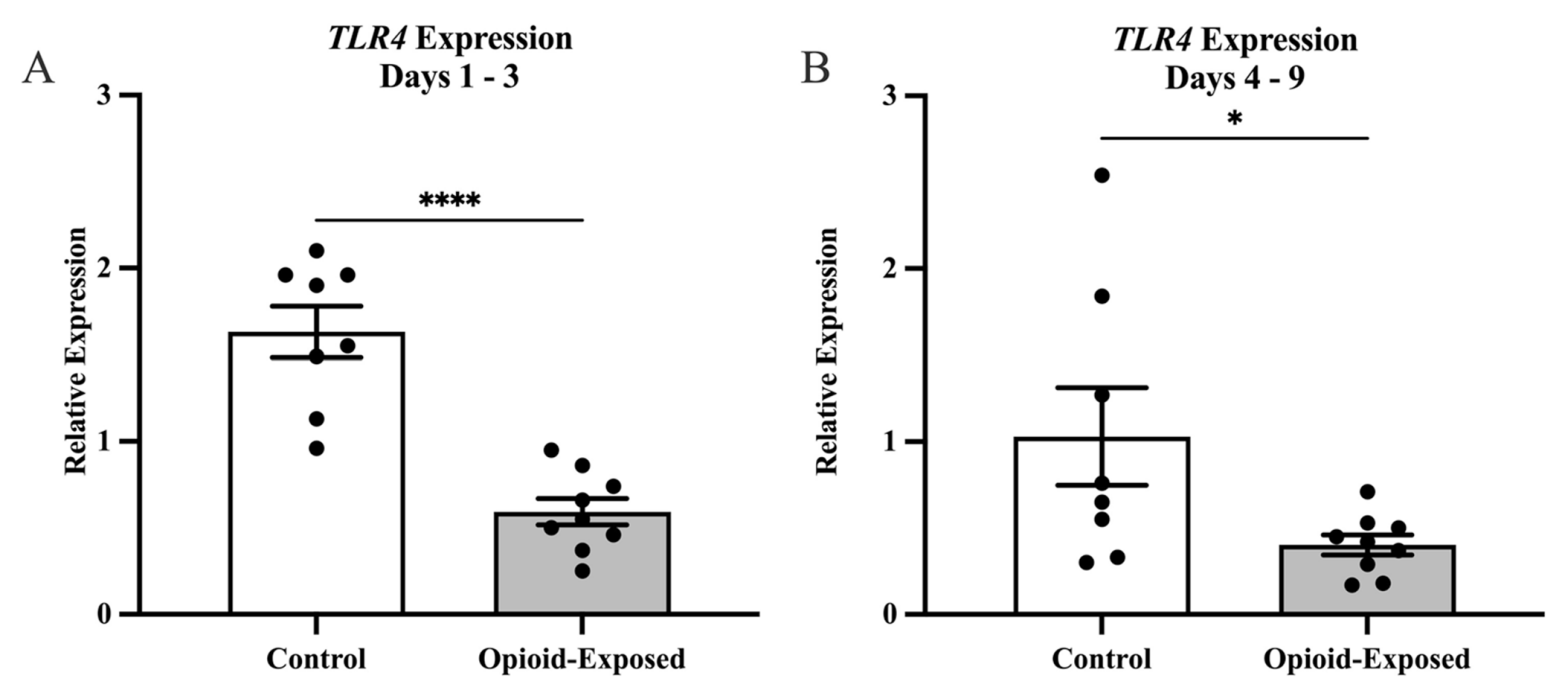
Expression of *TLR4* is significantly reduced in the stool of opioid-exposed neonates with NOWS. Expression of *TLR4* was measured in the stool of opioid-exposed (*n* = 9) and non-exposed control (*n*= 8) infants between days 1–3 (**A**) and days 4–9 (**B**) after delivery. Bars represent mean ± SEM. Data points indicate *TLR4* expression for one infant during the indicated time frame. *P < 0.05, ****P < 0.0001 via Mann-Whitney *U* test for relative gene expression levels compared between opioid-exposed and non-exposed infants.

**Fig. 3:**
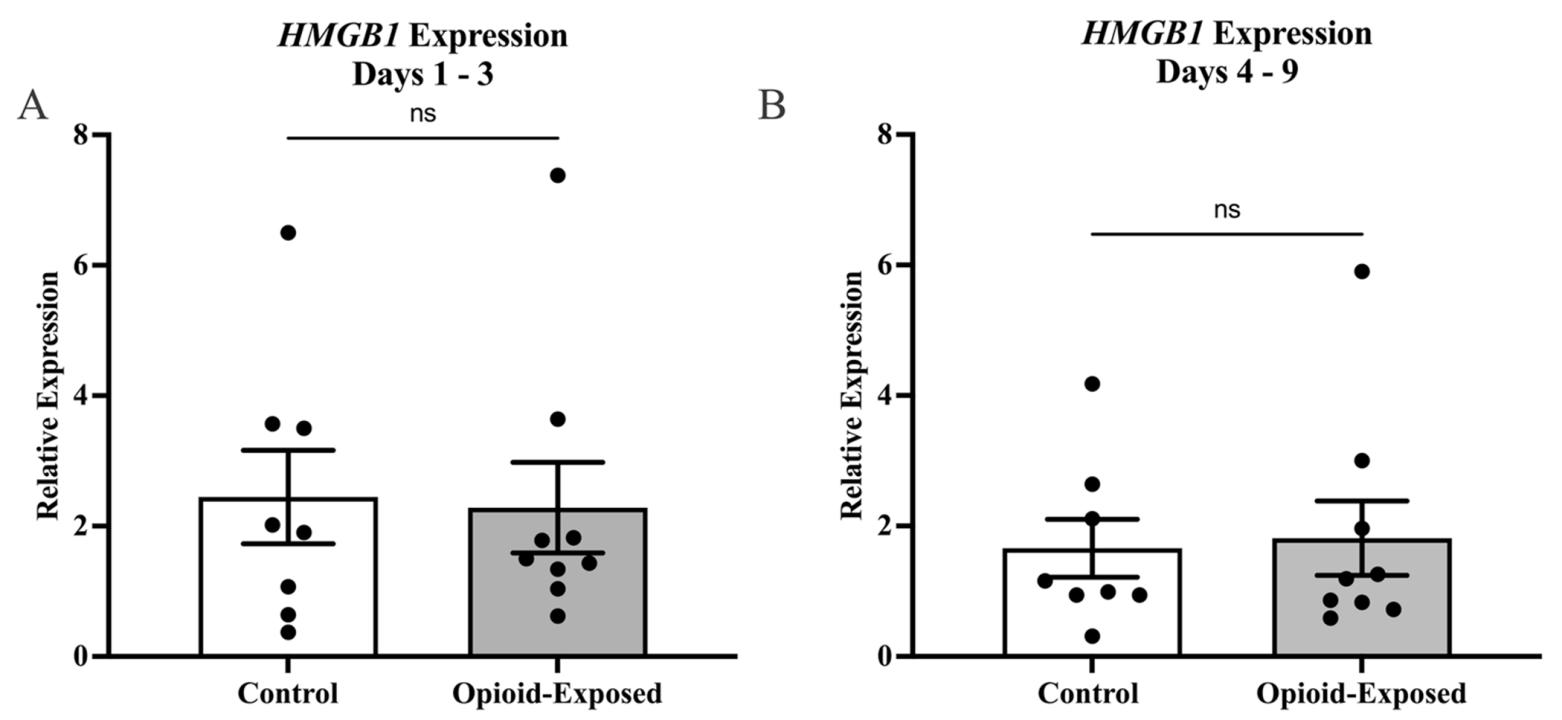
Expression of *HMGB1* is not significantly different in the stool of control and opioid-exposed neonates with NOWS. Expression of *HMGB1* was measured in the stool of opioid-exposed (*n* = 9) and non-exposed control (*n* = 8) infants between days 1–3 (**A**) and days 4–9 (**B**) after delivery. Bars represent mean ± SEM. Data points indicate *HMBG1* expression for one infant during the indicated time frame. ns =*P* > 0.05 via Mann-Whitney *U* test for relative gene expression levels compared between opioid-exposed and non-exposed infants in both time frames.

In order to determine the impact of in utero opioid exposure on the neonatal intestinal inflammatory milieu, we compared gene expression in stool samples from the opioid-exposed and non-exposed neonates between days 1 and 3 and between days 4 and 9 after delivery. We found that the relative expression of *TLR4* was significantly lower in the stool of the opioid-exposed infants between days 1 and 3 (*P* < 0.0001) and days 4 and 9 (*P* < 0.05) after delivery (Fig. 2). In contrast, we did not detect any significant differences in stool *HMGB1* expression in these infants during either time frame (Fig. 3).

**Fig. 4:**
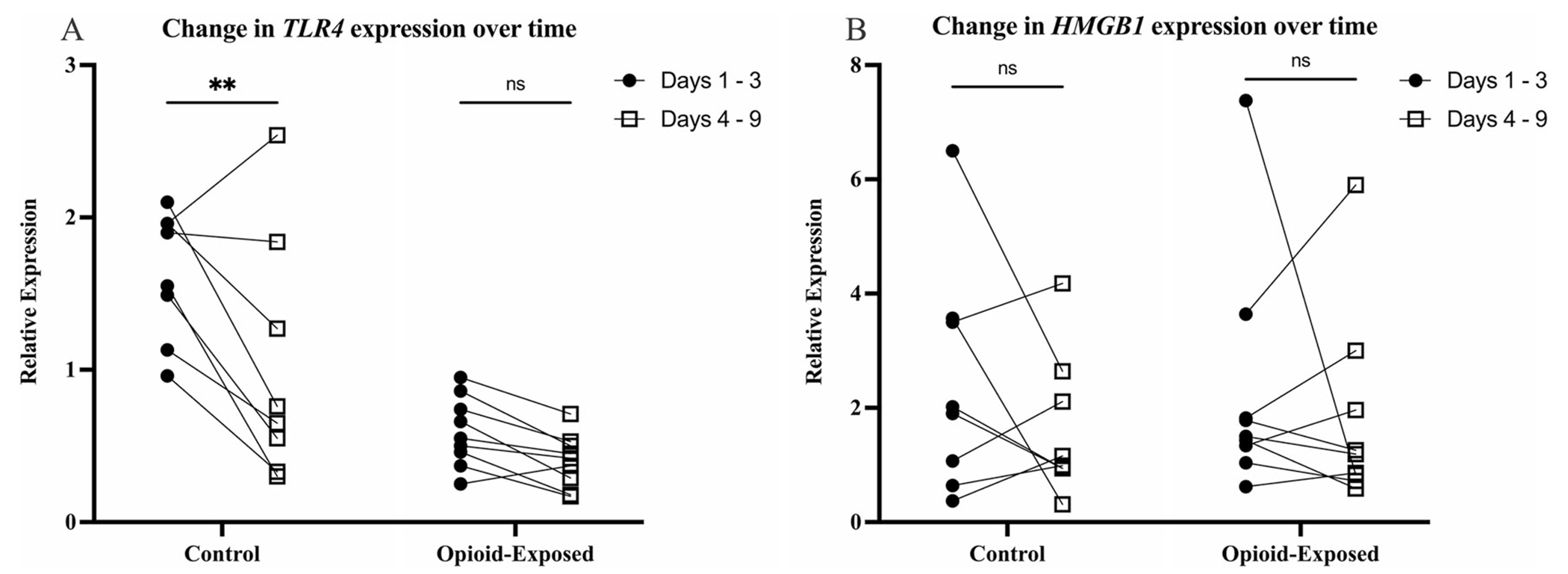
Expression of *TLR4* significantly decreases over time in the stool of infants in the control group. Expression of *TLR4* (**A**) and *HMGB1* (**B**) was measured in the stool of opioid-exposed (*n* = 9) and non-exposed control (*n* = 8) infants between days 1–3 and days 4–9 after delivery. Data points indicate gene expression for a single infant. Connected dots represent the same infant at two different time points. *P < 0.01, ns =*P* > 0.05 via Two-Way repeated measures ANOVA.

We subsequently analyzed the change in gene expression over time for individual patients. We found that there was a significant decrease in *TLR4* expression in the stool of the non-opioid exposed control infants between the samples collected on days 1–3 and days 4–9 (*P* < 0.01, Fig. 4A). In contrast, there was no significant difference between these time frames for the opioid-exposed infants (*P* > 0.05). There was also no clear pattern of stool gene expression changes for *HMGB1* for either treatment group (Fig. 4B).

## DISCUSSION

In this study, we demonstrate that expression of *TLR4*, but not *HMGB1*, is significantly decreased in the stool of a cohort of neonates exposed to opioids in utero. Although our initial hypothesis was that neonates with NOWS would exhibit increased *TLR4* expression, which could predispose them to NEC [5], our data suggest otherwise.

TLR4 is an innate pattern recognition receptor that recognizes LPS expressed by Gram-negative bacteria [18, 19] and has been linked to the development of NEC in preterm neonates [14]. Intestinal epithelial cell expression of TLR4 is required for the development of NEC in mice, and increases inflammatory cytokine production, accentuates epithelial barrier dysfunction, and promotes intestinal epithelial cell death during experimental NEC [14, 25–28]. It is hypothesized that TLR4 activation by a dysbiotic microbiome within the intestine of preterm neonates induces an unrestrained inflammatory response that leads to intestinal injury and NEC. The human intestine is colonized by trillions of microbes, and intestinal immunity must exist in a fine balance of responding to potentially harmful pathogens versus inducing injury through off target inflammatory responses to commensal microbes. This is particularly complex in preterm neonates where the intestinal immune system is immature, and the microbiome lacks the predominance of commensal species such as *Bifidobacterium* and *Lactobacillus* spp., observed for breastfed term neonates [29–31]. Additionally, an increased abundance of bacteria in the Gamma-proteobacteria class has been shown to precede the development of NEC [32, 33]. These data point to an important interplay between TLR4 activation and inflammation in the preterm neonatal intestine; however, the essential factors in the initiation and modulation of the inflammatory cycle in NEC remain areas of active research.

The significant decrease in TLR4 expression in the setting of in utero opioid exposure and NOWS demonstrates a potential role for opioids in modulating the intestinal inflammatory milieu in neonates. While is possible that postnatal opioid exposure impacts the expression of *TLR4*, this is unlikely to explain all of the observed effects given that *TLR4* levels were significantly lower in the stool of opioid-exposed neonates compared to non-exposed infants in the initial sample collection on Days 1–3 (Fig. 2). For four of the nine opioid-exposed infants, this collection was before treatment for NOWS was initiated, and one of the neonates was not treated for NOWS (Table 2). The physiologic importance of and mechanisms behind these observations remains to be determined, although there is a body of literature documenting the interplay between opioids and immunity, with a potential opioid-mediated immunosuppressive effect [34, 35]. In addition, the predominant role of gastrointestinal symptoms in NOWS supports an important impact of opioids on intestinal function in neonates, although the precise mechanisms and the role of inflammation in this process are unknown. There may also be important systemic effects from these gastrointestinal processes, as the inflammatory crosstalk involving the gut-brain axis could potentially mediate the association of opioid exposure with adverse neurodevelopmental outcomes [2, 36].

Importantly, this study is the first to investigate *TLR4* and *HMGB1* expression in the stool of opioid-exposed human neonates. In addition, we demonstrate that prospective acquisition of stool samples from a cohort of healthy neonates can be used to determine host intestinal gene expression patterns despite the abundance of bacterial genes in these samples. There is a paucity of data examining stool gene expression in samples from neonates, and this represents a potentially powerful technique that can non-invasively yield insights into the intestinal environment over the course of development [37–41].

Strengths of this study include that the opioid-exposed and non-opioid-exposed control infants were well-matched in baseline characteristics. Importantly, all of the infants were formula fed, which removed the significant confounding effect of variations in receipt of breast milk volumes. Other strengths include the availability of detailed information regarding the in utero exposure of each infant and their treatment for NOWS. Finally, we were fortunate to perform a longitudinal analysis of the markers of interest, which was provided by collecting stool samples at multiple time points. Limitations of this study include the small sample size, the inclusion of infants from a single center, and the analysis of a limited number of genes. Additionally, none of the neonates in this study developed NEC, so it is possible that gene expression patterns differ between neonates with NOWS who go on to develop NEC and those who do not. Given the rarity of NEC in this population, a prohibitively large sample size would be needed to test this hypothesis. Moreover, stool gene expression patterns are likely reflective of sloughed intestinal epithelial cells and may not fully represent gene expression patterns within the intestinal tissue itself; however, we have previously found that DNA methylation patterns are highly concordant between intestinal tissue samples and stool samples in neonates with and without NEC [42].

In conclusion, we have found *TLR4* expression in the stool of opioid-exposed infants is significantly decreased compared to non-exposed infants at days 1–3 and day 4–9. Thus, elevated *TLR4* expression in stool does not appear to explain the higher risk of NEC in opioid-exposed infants with NOWS, but these data do indicate that opioids may have a significant impact on the intestinal inflammatory milieu of neonates. Further studies are needed to evaluate the association of in utero opioid exposure and NEC and the impact of opioids on intestinal inflammation and development in neonates.

## Figures and Tables

**Table 1.** Baseline maternal and neonatal characteristics of cohort.

	Non-Exposed Neonates (*n* = 8)	Opioid-Exposed Neonates (*n* = 9)	*P* value
Maternal Age, mean, (SD), years	26.4 (2.7)	30.8 (5.4)	0.09
Male sex, No. (%)	2 (25)	4 (44)	0.62
Gestational Age, mean, (SD), wks	38.0 (1.3)	39.1 (0.7)	0.04
Birth weight, mean, (SD), kg	3.17 (0.51)	3.13 (0.40)	0.98
Treatment for NOWS, No. (%)	0 (0)	8 (89)	N/A

Data are presented as mean (standard deviation, SD) unless otherwise indicated.

Significance was determined via Mann-Whitney *U* test or Fischer’s exact test.

**Table 2.** Study population details.

Group	Maternal Age (Years)	Gestational Age (Weeks)	Birth Weight (kg)	Sex	Maternal Utox Results	Cord Tox Results	In Utero Opioid Exposure	NOWS Treatment	Age of Initial NOWS Treatment (Days)	Age at First Stool Collection (Days)	Age at Second Stool Collection (Days)
Control											
	23	38 6/7	3.0	Male	Negative	N/A	None	None	N/A	2	4
	23	36 2/7	2.9	Female	THC	N/A	None	None	N/A	2	8
	25	37 4/7	2.8	Female	Negative	N/A	None	None	N/A	2	4
	26	36 2/7	3.4	Female	Negative	N/A	None	None	N/A	3	5
	28	40 0/7	3.1	Female	Negative	N/A	None	None	N/A	2	5
	31	37 5/7	3.0	Female	Negative	N/A	None	None	N/A	2	5
	27	39 0/7	2.8	Female	Negative	N/A	None	None	N/A	1	7
	28	37 0/7	4.0	Male	Negative	N/A	None	None	N/A	1	4
Opioid-Exposed											
	29	39 1/7	3.4	Female	Buprenorphine	Negative	Buprenorphine	Methadone, Clonidine	3	2	7
	33	39 2/7	4.0	Male	Buprenorphine, TCA	Buprenorphine	Buprenorphine	Methadone, Clonidine	4	2	6
	36	37 5/7	2.4	Female	Buprenorphine, TCA^a^	Fentanyl, Opiates, Morphine	Buprenorphine	Methadone	2	3	8
	25	39 3/7	2.7	Female	Methadone	Methadone	Methadone	Methadone, Clonidine, Phenobarbital	1	2	5
	28	39 6/7	3.6	Male	Buprenorphine	Buprenorphine	Buprenorphine	Methadone	1	2	5
	24	40 0/7	3.5	Male	Buprenorphine, THC, TCA	Buprenorphine, THC	Buprenorphine	Methadone, Clonidine	3	2	4
	27	38 3/7	2.8	Male	Buprenorphine	N/A	Buprenorphine	Methadone, Clonidine	1	1	6
	36	39 0/7	2.8	Female	Methadone, TCA	Methadone, Cocaine	Methadone, Heroin	Methadone	3	2	9
	39	39 1/7	3.2	Female	Buprenorphine, THC	Buprenorphine, THC	Buprenorphine	None	N/A	2	6

The specific characteristics of mothers and infants enrolled in this study are provided for the control and opioid-exposed groups.

*THC* tetrahydrocannabinol, *TCA*+ suspected cross-reactivity with Flexeril or Vistaril, *N/A* not applicable.

a Unable to test for fentanyl due to reagent shortage.

## Data Availability

All datasets are immediately available upon request to the corresponding author.
